# Cholesterol-Dependent Cytolysins Produced by Vaginal Bacteria: Certainties and Controversies

**DOI:** 10.3389/fcimb.2019.00452

**Published:** 2020-01-10

**Authors:** Milda Pleckaityte

**Affiliations:** Laboratory of Immunology and Cell Biology, Institute of Biotechnology, Life Sciences Center, Vilnius University, Vilnius, Lithuania

**Keywords:** cholesterol-dependent cytolysin, vaginolysin, inerolysin, *Gardnerella* spp., *Lactobacillus iners*, pore-forming mechanism, virulence factors, bacterial vaginosis

## Abstract

Bacterial vaginosis (BV) is a vaginal anaerobic dysbiosis that affects women of reproductive age worldwide. BV is microbiologically characterized by the depletion of vaginal lactobacilli and the overgrowth of anaerobic bacterial species. Accumulated evidence suggests that *Gardnerella* spp. have a pivotal role among BV-associated bacteria in the initiation and development of BV. However, *Gardnerella* spp. often colonize healthy women. *Lactobacillus iners* is considered as a prevalent constituent of healthy vaginal microbiota, and is abundant in BV. *Gardnerella* spp. and *L. iners* secrete the toxins vaginolysin (VLY) and inerolysin (INY), which have structural and activity features attributed to cholesterol-dependent cytolysins (CDCs). CDCs are produced by many pathogenic bacteria as virulence factors that participate in various stages of disease progression by forming lytic and non-lytic pores in cell membranes or via pore-independent pathways. VLY is expressed in the majority of *Gardnerella* spp. isolates; less is known about the prevalence of the gene that encodes INY. INY is a classical CDC; membrane cholesterol acts a receptor for INY. VLY uses human CD59 as its receptor, although cholesterol remains indispensable for VLY pore-forming activity. INY-induced damage of artificial membranes is directly dependent on cholesterol concentration in the bilayer, whereas VLY-induced damage occurs with high levels of membrane cholesterol (>40 mol%). VLY primarily forms membrane-embedded complete rings in the synthetic bilayer, whereas INY forms arciform structures with smaller pore sizes. VLY activity is high at elevated pH, which is characteristic of BV, whereas INY activity is high at more acidic pH, which is specific for a healthy vagina. Increased VLY levels in vaginal mucosa *in vivo* were associated with clinical indicators of BV. However, experimental evidence is lacking for the specific roles of VLY and INY in BV. The interplay between vaginal bacterial species affects the expression of the gene encoding VLY, thereby modulating the virulence of *Gardnerella* spp. This review discusses the current evidence for VLY and INY cytolysins, including their structures and activities, factors affecting their expression, and their potential impacts on the progression of anaerobic dysbiosis.

## Introduction

Vaginal bacterial species composition and abundance were compared in reproductive-aged women (Ravel et al., 2011; Fettweis et al., 2014), and the results revealed that *Lactobacillus*-dominated microbial communities are the hallmark of a healthy vagina (Ma et al., 2012; Vaneechoutte, 2017a). Otherwise healthy asymptomatic women harboring a polymicrobial mixture of anaerobic bacteria with few lactic acid-producing lactobacilli also represents a healthy vagina (Ma et al., 2012; Smith and Ravel, 2017), although the lower numbers of lactobacilli reduce their protection against pathogenic microorganisms (Anahtar et al., 2015). High-throughput sequencing and microscopy studies to classify vaginal species provide a better understanding of clinical conditions associated with the disturbance of healthy, *Lactobacillus*-dominated microbiota, which lead to bacterial vaginosis (BV) (Nugent et al., 1991; Srinivasan et al., 2012) and the recently identified clinical condition called aerobic vaginitis (Donders et al., 2017).

BV is the vaginal condition associated with poor reproductive and obstetric sequelae (Kenyon et al., 2013; van de Wijgert and Jespers, 2017). BV is microbiologically characterized by the depletion of most vaginal *Lactobacillus* species and the overgrowth of diverse anaerobes (Srinivasan and Fredricks, 2008; Huang et al., 2014; Onderdonk et al., 2016). Intensive DNA-based studies on vaginal microbiota did not identify the etiology of BV and suggest that BV is a complex condition that may involve several different diseases (Cerca et al., 2017). This hypothesis was proposed because the primary causative pathogen(s) of BV have not been unambiguously determined (Muzny et al., 2019). Further, epidemiological data suggest that BV may be sexually transmitted (Fethers et al., 2008; Swidsinski et al., 2014).

Deep sequencing showed that BV is associated with an array of anaerobic bacteria (Zozaya-Hinchliffe et al., 2010; Srinivasan et al., 2012). Facultative anaerobic bacterial species of the genus *Gardnerella* have been recovered from vaginal samples of almost all women with BV (Fredricks et al., 2007; Srinivasan et al., 2012). *Gardnerella* spp. have higher virulence potential than other BV-associated bacteria, thereby supporting its possible role in BV development (Patterson et al., 2010; Muzny and Schwebke, 2013; Robinson et al., 2019). The key feature of BV is the presence of a bacterial biofilm on vaginal epithelial cells, which predominantly consists of *Gardnerella* spp. and other incorporated bacterial groups (Swidsinski et al., 2005, 2014). The impact of neighboring BV-associated bacteria has been analyzed for effects on BV pathogenesis (Muzny et al., 2018; Castro et al., 2019; Gilbert et al., 2019). However, the pivotal role of *Gardnerella* in BV has been debated because of its presence in the healthy vagina. There are likely differences in virulence among *Gardnerella* strains, and the expression of virulence traits may increase under certain conditions (Hickey and Forney, 2014; Janulaitiene et al., 2018; Castro et al., 2019; Muzny et al., 2019).

*Lactobacillus iners* is another bacterium found at high levels in healthy and BV-positive women (Srinivasan et al., 2012; Petrova et al., 2015), although the hallmark of BV is a depletion of lactobacilli. *L. iners*-dominated microbiota are often detected at the transitional stage between normal and BV conditions (Petrova et al., 2017; Vaneechoutte, 2017b) and during menses (Santiago Lopes dos Santos et al., 2012); therefore, these microbial populations are considered to be less stable. *L. iners* predominantly produces L-lactic acid, which has lower protective capacity than the D-lactic acid released by other vaginal lactobacilli (Witkin and Linhares, 2017). The adaptation of *L. iners* to different environmental conditions may be due to the repertoire of gene expression, which ensures competitive adaptability and survival (Macklaim et al., 2011; France et al., 2016). Different *L. iners* lineages or groups with different adaptive properties may have functional roles in this adaptation (Petrova et al., 2017).

Both *Gardnerella* spp. and *L. iners* are present in the healthy human vagina and during anaerobic dysbiosis, and both secrete cholesterol-dependent cytolysins (CDCs) that belong to a common family of pore-forming toxins (PFTs) (Alouf, 2003; Christie et al., 2018). CDCs are produced by many pathogenic Gram-positive bacteria, and are recognized as virulence factors that participate in various stages of disease progression (Los et al., 2013). CDCs are secreted by a few Gram-negative bacteria that inhabit anaerobic soils and do not colonize humans or animals (Hotze et al., 2013). CDCs are cytotoxic to eukaryotic cells, including erythrocytes, and are known as cytolysins or hemolysins. *L. iners* secretes the CDC toxin inerolysin (INY) (Rampersaud et al., 2011), whereas *Gardnerella* spp. secrete the CDC toxin vaginolysin (VLY) (Rottini et al., 1990; Cauci et al., 1993; Gelber et al., 2008). VLY-mediated cell lysis is visible by cultivation of *Gardnerella* spp. on solid agar supplemented with human blood; β-hemolysis surrounding colonies indicates complete lysis of erythrocytes in the medium. The effect of these CDC toxins could be prerequisite for the survival and adaptation of *Gardnerella* and *L. iners* under diverse environmental conditions and during the development of BV. This review discussed the current knowledge, recent evidence, and implications relating to VLY and INY.

## Genetic Characteristics

The cytolysin-coding genes *vly* and *iny* are single-copy genes in the chromosomes of *Gardnerella* spp. and *L. iners*, respectively. Both genes were cloned and sequenced (Gelber et al., 2008; Rampersaud et al., 2011). Among *Gardnerella* spp. isolates, VLY exhibits higher amino acid sequence variations, especially in the N-terminal region, than other CDCs. However, the detected amino acid substitutions did not affect the cytolytic activity of VLY (Pleckaityte et al., 2012). The VLY protein is 56 kDa, and INY protein is 57 kDa. Both toxins contain a signal sequence at the N-terminus that ensures secretion via a type II secretion pathway (Tweten, 2005). A recent study reported that VLY was detected in extracellular vesicles (EVs) produced by the *Gardnerella vaginalis* ATCC 14019 strain (Shishpal et al., 2019). EV-mediated delivery of toxins and other virulence factors has been identified for many pathogenic Gram-positive bacteria (Brown et al., 2015; Liu et al., 2018). It is unclear whether the majority of VLY is secreted via EVs and how this pathway promotes *Gardnerella* spp. survival, colonization, and pathogenesis.

Genetic and phenotypic heterogeneity in the genus *Gardnerella* enabled the identification of genomic subgroups (Ahmed et al., 2012; Balashov et al., 2014; Schellenberg et al., 2016). Multiple *Gardnerella* subgroups were detected in non-cultured vaginal samples. *Gardnerella* multigroup communities were positively associated with BV (Balashov et al., 2014; Janulaitiene et al., 2017; Shipitsyna et al., 2019). A recent study identified and described 13 new species within the genus *Gardnerella*, including *Gardnerella leopoldii, Gardnerella swidsinskii, Gardnerella piotii*, and *G. vaginalis* (Vaneechoutte et al., 2019). It is likely that species or genomic subgroups are specifically associated with BV due to differences in their virulence potential. In our study (Janulaitiene et al., 2018), the majority of isolates of subgroup 4 that were associated with healthy microbiota produced a low amount of VLY *in vitro* compared to other groups. We identified isolates of subgroup 2 that were the *vly-*negative. Isolates with and without the *vly* gene colonized the same woman (Janulaitiene et al., 2018). By contrast, *iny*-negative isolates have not been identified, and INY was detected in all tested supernatants of cultured *L. iners* strains (Rampersaud et al., 2011).

## Structural Features of CDC Proteins and an Overview of the Pore-Forming Mechanism

**Figure 1:**
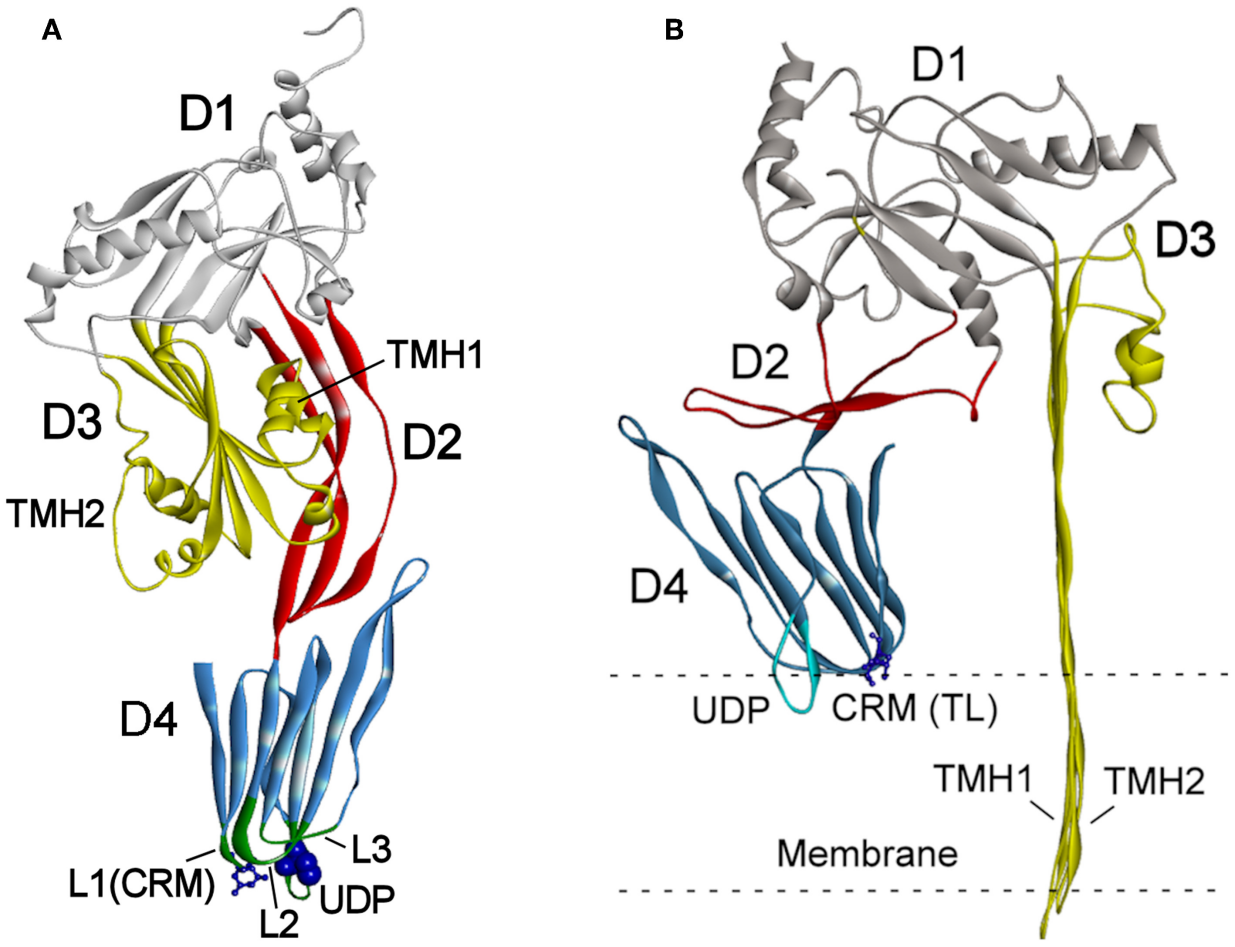
**(A)** Schematic presentation of the crystal structure of PLY (PDB ID: 4QQA; Park et al., 2016). **(B)** One subunit of oligomeric PLY inserted into a membrane in the cryoEM structure (PDB ID: 5LY6; van Pee et al., 2017). The features of the structure are marked as follows: domains D1–D4 (highlighted in different colors), loops L1–L3, undecapeptide (UDP) in D4, the two-residue (Thr-Leu pair shown as sticks) cholesterol recognition motive (CRM) in loop L1, and the Cys residue in UDP (shown as blue spheres). The transmembrane hairpins TMH1 and TMH2 of D3 span the membrane in **(B)**.

The structures of CDCs from various bacterial genera were determined and revealed a four-domain structural organization (Rossjohn et al., 1997; Polekhina et al., 2004; Lawrence et al., 2015, 2016) (Figure 1A). The key sequence motifs involved in the recognition of cholesterol and membrane binding are highly conserved across all known CDCs.

**Figure 2:**
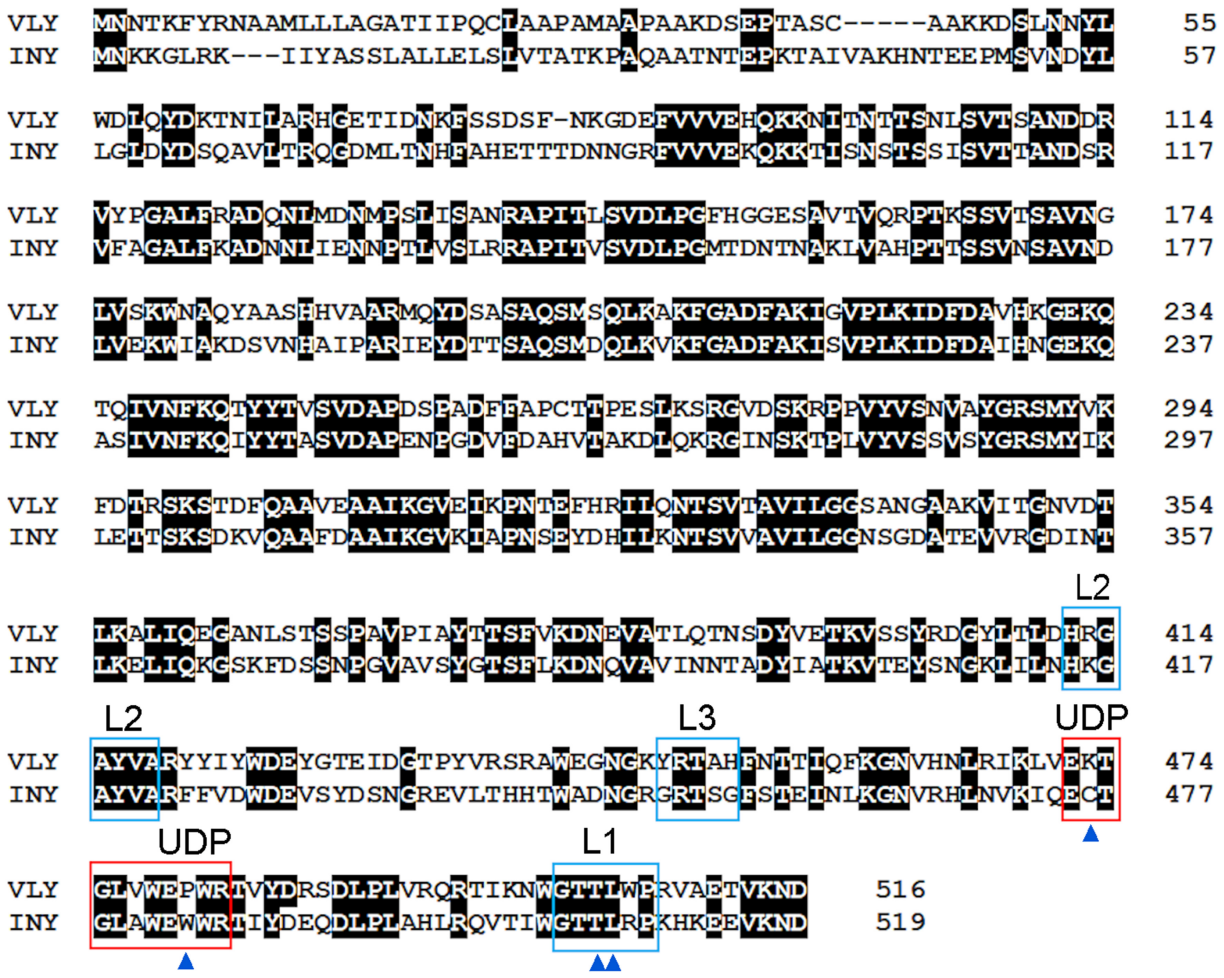
Amino acid sequence alignment of VLY (accession no. ACD63042) and INY (accession no. WP_006730404). The sequences were aligned using Clustal W (Aiyar, 2000). Strictly conserved residues are indicated with a black background. Loops L1–L3 and the undecapeptide (UDP) are framed in blue and red, respectively. Cys residue in UDP of INY, the Pro/Trp in UDP, and the Thr-Leu pair (CMR) in L1 are indicated by blue triangles.

**Figure 3:**
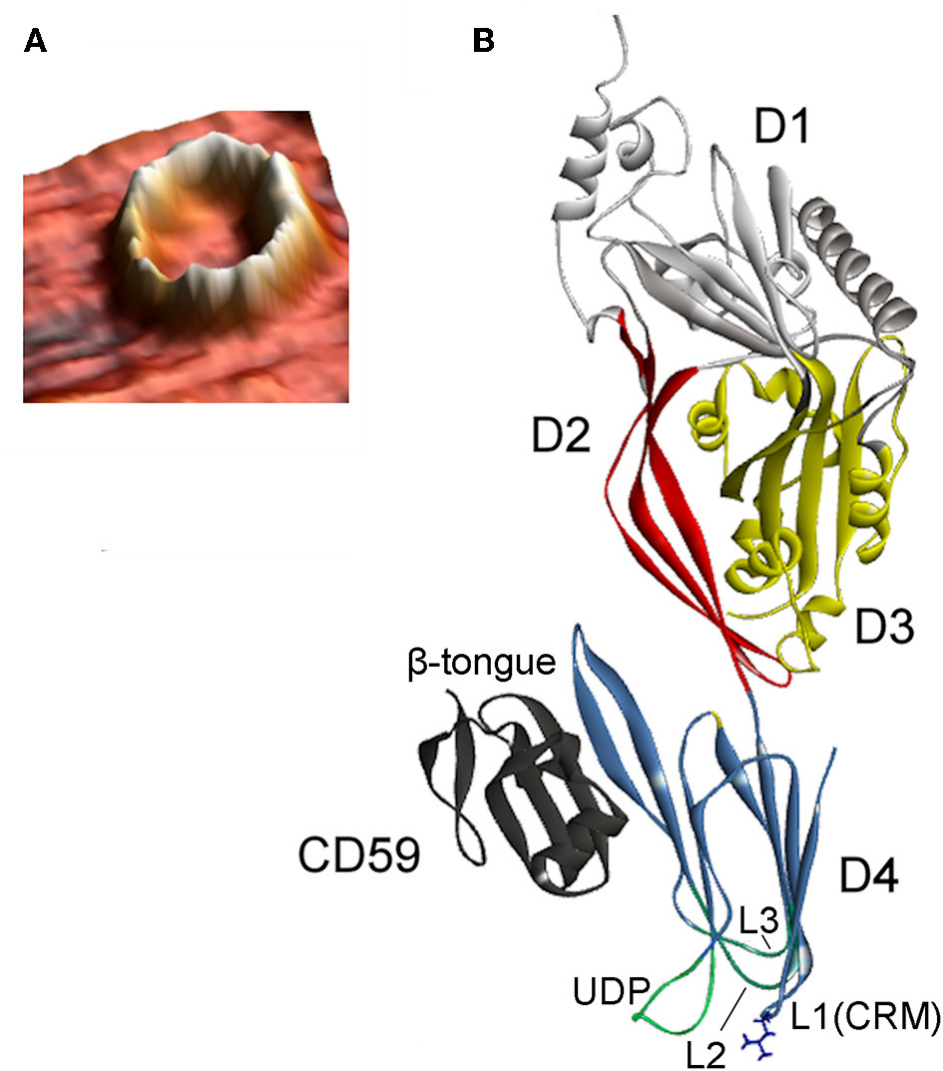
**(A)** Atomic force microscopy three-dimensional topography image of the oligomeric structure obtained after reconstitution of VLY into a cholesterol-rich artificial bilayer. Calculated protrusion of the ring-shaped defect above the membrane is consistent with the height of inserted pore (reproduced with permission from Ragaliauskas et al., 2019). **(B)** Crystal structure of VLY in complex with hCD59 (PDB ID: 5IMY; Lawrence et al., 2016). VLY interacts with hCD59 via the β-tongue located in D4, similarly as ILY. Loop L1 contains the Thr-Leu (TL) pair of CRM.

The mechanisms of CDC interaction with membranes and pore formation have been extensively studied for perfringolysin (PFO) from *Clostridium perfringens* (Rossjohn et al., 1997; Ramachandran et al., 2002) and pneumolysin (PLY) from *Streptococcus pneumoniae* (Gilbert et al., 1998; Vögele et al., 2019). Cytolysins are secreted as water-soluble monomers (Tweten, 2005), which recognize membrane cholesterol via the cholesterol recognition motive (CRM) composed of the Thr-Leu pair in the L1 loop of domain D4 (Farrand et al., 2010) (Figure 1A). Recognition of cholesterol triggers the insertion of D4 structural components, an 11 amino acid undecapeptide (UDP) and the nearby L2–L3 loops, into a membrane that provides anchorage and stability of the monomer (Dowd et al., 2012; Christie et al., 2018). The conservative UDP sequence in the majority CDCs, including INY, contains a Cys residue (Figure 2) that, in the reduced state, ensures maximal activity of toxins (Tweten, 2005; Rampersaud et al., 2011). Therefore, CDCs are also known as thiol-activated cytolysins. After membrane binding, structural changes occur in the protein that enable monomer-monomer interactions. The monomers oligomerize into rings and form a prepore structure on the membrane surface. The prepore is an SDS-resistant oligomeric complex characterized by a dense structure of ordinated and correctly oriented monomers. The prepore does not perforate the membrane (Hotze et al., 2002; Ramachandran et al., 2005). The transition from prepore to pore is accompanied by dramatic structural changes in domain D3, leading to the conversion of two helix bundles of each monomer to transmembrane β-hairpins (TMH) (Hotze and Tweten, 2012). Bilayer perforation is mediated by insertion of the TMH regions, and requires the helices to closely approach a membrane (Figure 1B). This is achieved by rotation of domain D2 following disruption of the D3 interface (Czajkowsky et al., 2004; Leung et al., 2014; van Pee et al., 2017). The β-barrel of the membrane-embedded pore complex (Figure 3A) is hydrophobic on the outside and hydrophilic inside (van Pee et al., 2017; Christie et al., 2018). This structure favors the displacement of membrane lipids and stimulates water influx, which finalizes the formation of a water-filled pore (Vögele et al., 2019).

## Characteristics of Vaginolysin and Inerolysin Activities

### Cholesterol Is Not a Single CDCs' Receptor: The Vaginolysin Case

Cholesterol embedded into a cell membrane has been identified as a common and indispensable binding target for the majority CDCs (Hotze and Tweten, 2012). A distinct group of CDCs primarily bind to human complement glycoprotein CD59 (hCD59) rather than to cholesterol. Three members of this distinct group include VLY, *Streptococcus intermedius* intermedilysin (ILY), and *Streptococcus mitis* lectinolysin (Giddings et al., 2004; Gelber et al., 2008; Wickham et al., 2011). The glycosylphosphatidylinositol (GPI)-anchored human cell-surface receptor hCD59 interacts with the membrane attack complex (MAC) components C8α and C9, thereby blocking the accidental activity of MAC (Iacovache and van der Goot, 2004; Huang et al., 2007). Thus, hCD59 which ensures the unwanted lysis of host cells due to the activity of complement, serves as a receptor for some bacterial CDCs. Wickham et al. (2011) investigated ILY and MAC binding to hCD59; mutation of amino acid residues in hCD59 that modulate binding to ILY affects its protection against MAC-mediated lysis. The authors proposed that the existence of hCD59-responsive toxins may indicate microbial coevolution with humans, as the primary role of hCD59 is to block unwanted lysis of host cells.

The pore-forming mechanism of hCD59-dependent cytolysins is best-studied for ILY (LaChapelle et al., 2009; Farrand et al., 2010). When encountering host cells, ILY binds to hCD59 via the β-tongue located in D4 (Figure 3B). This event triggers the same structural changes as that of non-hCD59-dependent CDCs, but a pore does not form due to the lack of cholesterol. Contact between ILY and hCD59 allows the CRM-cholesterol interaction that triggers insertion of the L1–L3 loops into the bilayer (Farrand et al., 2010; Hotze and Tweten, 2012). After ILY is anchored to the membrane via the inserted loops, it disengages from hCD59 during the transition from a prepore to a pore. The release of hCD59 favors structural changes that are necessary for pore formation, as the increased binding affinity between ILY and hCD59 slows the rate of the prepore to pore transition and reduces cell lysis (Wickham et al., 2011). Boyd et al. (2016) recently reported that ILY interaction with hCD59, rather than with cholesterol, induced structural changes leading to collapse of the prepore structure, whereas cholesterol was required only for the final membrane perforation stage.

VLY-mediated cytotoxic activity was detected on cells that expressed hCD59 on their surface (Gelber et al., 2008; Zilnyte et al., 2015). Antibodies against VLY inhibited its cytolytic activity, demonstrating the specificity of VLY interaction with the cells (Zvirbliene et al., 2010; Pleckaityte et al., 2011). Membrane cholesterol is indispensable for the pore-forming activity of VLY and other known hCD59-dependent CDCs (Giddings et al., 2004; Gelber et al., 2008). ILY is a human-specific cytolysin (Giddings et al., 2004). By contrast, the cells lacking hCD59 were susceptible to VLY-mediated lysis, albeit at the elevated VLY concentrations (Zilnyte et al., 2015). Analysis of the crystal structure of VLY complexed to hCD59 (Figure 3B) revealed novel information regarding the dualistic activity of VLY (Lawrence et al., 2016). The stable VLY-hCD59 complex used for structural studies was obtained only with mutant VLY, which has limited capacity to oligomerize but retains its ability to interact with the receptor. The attempts of Lawrence et al. (2016) and our group (unpublished observations) to crystalize VLY alone were not successful.

The conformation of the L1 loop, which contains the CRM, is critical for cytolysin binding to cholesterol (Farrand et al., 2010; Lawrence et al., 2016). The structure of the UDP loop affects cholesterol-mediated membrane binding and D3 structural changes, which are the principal steps in the allosteric pore-formation pathway (Dowd et al., 2012). Superimposition of available CDC structures indicates that amino acids in the UPD loop of non-hCD59-dependent cytolysins interact with the L1 loop via hydrogen bonds, which leads to the proper conformation of L1 for initial binding with membrane cholesterol (Hotze and Tweten, 2012; Lawrence et al., 2016). The UDP loop in hCD59-dependent cytolysins contains Pro instead of Trp (Figure 2), which does not promote interaction of the neighboring amino acids with the L1 loop to ensure the proper orientation for cholesterol binding (Lawrence et al., 2016). Thus, hCD59-dependent cytolysins bind hCD59 instead of membrane cholesterol. Analysis of the two monomers in the VLY-hCD59 complex revealed that the UDP loop can adopt two conformations that are characteristic of either classical non-hCD59-dependent CDCs (e.g., PFO) or strictly hCD59-dependent ILY. The adopted UDP conformations predict which primary binding target, hCD59 or cholesterol, is selected in the host membrane to execute VLY pore-forming activity. It is likely that hCD59 is a more effective binding target than cholesterol for VLY interaction with the host membrane (Lawrence et al., 2016). INY is a classical non-hCD59-dependent cytolysin, although its primary amino acid sequence shows the greatest similarity to the CD59-dependent CDCs ILY (sequence identity 48.8%, Rampersaud et al., 2011) and VLY (sequence identity 50.8%, Figure 2).

### Membrane Binding

CDCs bind and affect membranes with high cholesterol levels, which contain a cholesterol threshold of >30 mol% of total membrane lipid (Bavdek et al., 2007; Johnson et al., 2012; Zilnyte et al., 2015). The accessibility of membrane cholesterol to CDCs has been a subject of debate. CDCs were proposed to bind to microdomains called lipid rafts, which are enriched with sphingolipids, cholesterol, and proteins (Waheed et al., 2001). However, sphingomyelin, which is an essential component of lipid rafts, inhibited PFO binding to a membrane (Flanagan et al., 2009). Subsequent studies showed that CDC binding required membrane conditions with surface-exposed cholesterol (Nelson et al., 2008). Cholesterol accessibility is modulated by the structures of phospholipid headgroups and acyl chains. Tightly packed phospholipids reduced cholesterol exposure, whereas loosely packed phospholipids enhanced cholesterol availability to CDCs (Rojko and Anderluh, 2015; Chakrabarti et al., 2017; Morton et al., 2019).

The results of electrochemical studies revealed that VLY binds to tethered lipid bilayer membranes (tBLMs) and impacts membrane integrity when cholesterol content was 50 mol% (Ragaliauskas et al., 2019), whereas other methods detected SDS-resistant VLY oligomeric complexes on liposomes containing cholesterol contents of 40–55 mol% (Zilnyte et al., 2015). The fact that VLY did not permeabilize cells in the apical side of a three-dimensional model of vaginal epithelium can be explained by the differential abundance of surface-associated hCD59 and the diverse cholesterol contents in the cell membranes (Garcia et al., 2019). By contrast, INY was active on synthetic membranes with low cholesterol contents of 20–30 mol%. INY binding to tBLMs and the extent of INY-induced membrane damage were directly dependent on membrane cholesterol concentration (Ragaliauskas et al., 2019). INY is likely to be the first CDC with activity on synthetic membranes that does not require a certain cholesterol threshold. However, INY-induced membrane damage was measured in bilayers with specific phospholipid compositions (Ragaliauskas et al., 2019).

Measuring the effects of the lipid environment on the activity of VLY and INY requires detailed investigations. PFO and streptolysin (SLO) have different binding specificities to the same natural and synthetic membranes (Farrand et al., 2015; Johnson et al., 2017). Altering the structure of the L3 loop by mutating critical residues changed the membrane binding parameters of PFO and SLO. CDCs may have evolved the capacity to select lipid environments by manipulating the L3 structure, and thereby display individual specificities for cholesterol (Farrand et al., 2015; Christie et al., 2018).

### Pore Size and Membrane Damage

**Figure 4:**
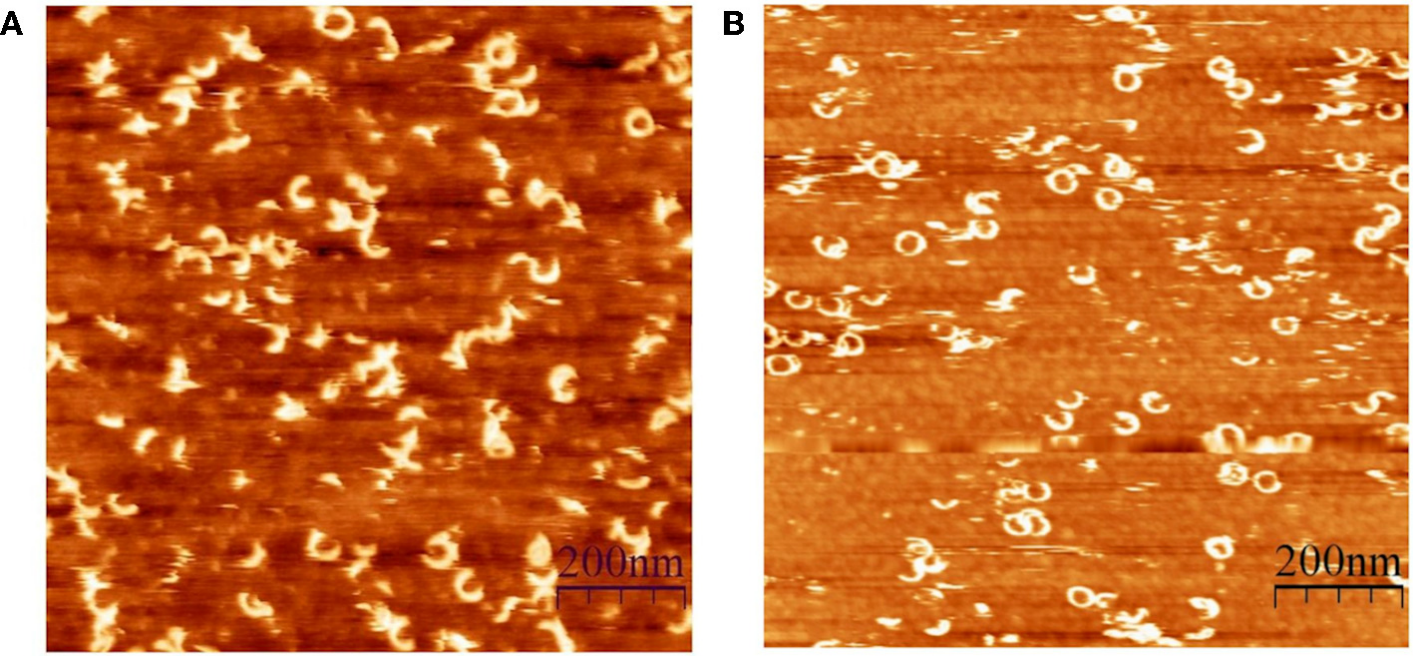
Atomic force microscopy topography images of the ring and arciform-shaped structures obtained after reconstitution of INY **(A)** and VLY **(B)** into tBLMs (reproduced with permission from Ragaliauskas et al., 2019).

CDCs belong to the β-PFT family because the β-sheet structures cross the host membrane (Figure 1). The distinguishing feature of the CDCs among β-PFTs is the large pore size; more than 30 monomers constitute the full rings, and pore diameters measure up to 30 nm (Hotze and Tweten, 2012). However, PLY (Sonnen et al., 2014), suilysin (Leung et al., 2014), ILY (Boyd et al., 2016), listeriolysin O (LLO) (Mulvihill et al., 2015), VLY, and INY (Ragaliauskas et al., 2019) also form incomplete rings, slits, and arcs that perforate synthetic membranes (Figure 4). Atomic force microscopy results indicated that arciform structures were predominate after reconstitution of INY into cholesterol-rich tBLMs (Figure 4A), whereas membrane-embedded complete rings with a diameter of 26 nm were predominate for VLY (Figure 4B) (Ragaliauskas et al., 2019). The majority of arciform structures produced by VLY and INY are inserted into the lipid bilayer and form a water-filled pore (Ragaliauskas et al., 2019). The VLY-generated pore geometries in tBLMs may differ from those in hCD59-containing membranes.

The arcs and slits that perforate membranes generate smaller pore sizes than those of complete rings. Pore size controls the molecules that can pass through the cell membrane, including ions, small molecules, and proteins (Dal Peraro and van der Goot, 2016). Presumably, pore size depends on the CDC function in the cell. LLO exhibits diverse activities in *Listeria monocytogenes* infection and triggers various host cell responses (Hamon et al., 2012). LLO generated small membrane perforations that leaked Ca^2+^ ions and protons from a eukaryotic cell (Shaughnessy et al., 2006), whereas other toxin activities in the host may generate extensive membrane damage (Osborne and Brumell, 2017). Another study concluded that pore size is not completely correlated with PFO- and SLO-mediated membrane damage (Ray et al., 2019). Los et al. (2013) observed differential effects of CDC-induced small and large pores *in vitro*; however, these differences were not evident in *in vivo* data. Pore-mediated membrane damage depends on CDCs and host responses to seal the pores and prolong cell survival (Cassidy and O'Riordan, 2013; Brito et al., 2019).

### Effect of pH

Several CDCs display pH-dependent activity, which is well-studied for LLO (Schuerch et al., 2005; Bavdek et al., 2012; Podobnik et al., 2015). LLO remains active at acidic pH, whereas it is irreversibly inactivated due to unfolding at neutral pH (Schuerch et al., 2005; Bavdek et al., 2012). The unfolding occurs in the D3 domain, and three acidic residues participate in the denaturation process (Schuerch et al., 2005). Podobnik et al. (2015) suggested that LLO forms pores irrespective of pH, but ionic conductance of the pores is regulated by a His residue that is pH-dependent. The pH-dependent activity of LLO may have biological significance. *L. monocytogenes* replicates within phagocytic host cells; therefore, the bacterium needs to escape from a vacuole that formed around it during entry to reach the host cell cytosol. *L. monocytogenes* acidifies the lumen of the vacuole, which activates LLO so that the cytolysin can destroy the vacuole membrane. LLO is not active at neutral pH, thereby preventing host cell destruction (Hamon et al., 2012; Osborne and Brumell, 2017).

INY and VLY effects on human erythrocytes and cholesterol-rich synthetic membranes is pH-sensitive. INY is active at acidic pH and inactive at neutral pH, whereas VLY activity peaks at neutral pH and is marginal at acidic pH (Rampersaud et al., 2011; Ragaliauskas et al., 2019). The pH- and temperature-dependent loss of activity is not related to protein denaturation, which is distinct from the dependence of LLO (Rampersaud et al., 2011; Ragaliauskas et al., 2019). INY regains activity after a pH shift from neutral to acidic (Rampersaud et al., 2011). Ratner et al. found that INY binding to membrane, including the oligomerization step, is pH-independent. The final step, INY membrane insertion, is impaired at neutral pH (Rampersaud et al., 2018). Ragaliauskas et al. (2019) reported that binding of INY and VLY to tBLMs is pH-sensitive. INY demonstrated weak or no binding activity to artificial lipid bilayer at neutral pH. Different INY and VLY activity profiles are consistent with vaginal pH under certain physiological conditions. INY is more active in the pH range characteristic of the healthy vagina, whereas VLY is active at higher pH specific for BV (Donders, 2007). The fact that INY is active in the pH range of 4.5–6.0 suggests that this toxin may be involved in the adaptation and survival of *L. iners* during transitional stages and dysbiosis.

### Cytolysin Concentration *in vivo*

There are some limitations in interpreting the results of mechanistic and functional studies of PFTs (Los et al., 2013), and these limitations should be considered for interpreting VLY and INY data in various models. Specifically, high doses of purified toxins used *in vivo* might not be relevant under physiological conditions (Los et al., 2013). The concentration of CDCs may be related to the ability to form pores in host membranes. High CDC doses, which are called lytic concentrations, are linked with the formation of lytic pores that lead to cell death. CDC doses at low, sublytic levels due to expression downregulation or lower bacterial densities is likely to produce non-lytic pores that lead to the modulation of cell signaling cascades (Ratner et al., 2006; Aguilar et al., 2009). CDCs at sublytic concentrations may affect host cells indirectly through pore-independent functions (Meehl and Caparon, 2004; Osborne and Brumell, 2017). However, CDC quantities under physiological conditions are unknown, except for PLY. Unexpectedly high median concentrations of PLY up to 30 μg/mL were detected in the cerebrospinal fluid (CSF) of patients who survived pneumococcal meningitis (Wall et al., 2012). Another study reported PLY concentrations up to 0.18 μg/mL in CSF of patients with meningitis (Spreer et al., 2003). Cell lysis *in vitro* was not visible at 0.5 μg/mL PLY, although the *in vivo* lytic capacity of PLY was enhanced by other factors (Spreer et al., 2003; Wippel et al., 2011).

In previous reports, VLY content was measured solely in planktonic cultures of various *Gardnerella* spp. isolates (Randis et al., 2009; Pleckaityte et al., 2012; Tankovic et al., 2017; Janulaitiene et al., 2018). Recently, VLY contents were measured in vaginal samples of women with healthy vaginal microbiota or anaerobic dysbiosis (Nowak et al., 2018). The median VLY concentrations were highest (3 ng/mL) in vaginal samples with deficient *Lactobacillus* (Nowak et al., 2018), intermediate concentrations in the presence of primarily *L. iners*, and lowest concentrations in the presence of primarily *L. crispatus*. The authors proposed that VLY might represent a marker of BV, as other characteristics of the vaginal samples also were associated with VLY concentrations, including *Gardnerella* spp. abundance, pH, and Nugent score (Nowak et al., 2018). The VLY concentrations in these vaginal samples (Nowak et al., 2018) were high enough to lyse human erythrocytes *in vitro*. VLY-mediated cytotoxicity to vaginal epithelial cells *in vitro* was detected at substantially higher concentrations (Zilnyte et al., 2015).

## Interpretations of the Physiological Roles of Inerolysin and Vaginolysin

There is a general concern that studies on the pathogenesis of human vaginal dysbiosis are challenging due to many factors, including the lack of a suitable animal model, vaginal microbiota are a dynamic community, and the impact of host and interplay between neighboring microorganisms. Studies on the role of cytolysins produced by vaginal bacteria have encountered similar difficulties. There are no methods for genetic manipulation of *Gardnerella* spp. remain a limitation. Cytolytic activity, mechanisms of pore formation, and some biological parameters have been studied using human cells, cell lines, and artificial bilayers. Recently, a three-dimensional polarized human vaginal tissue model has been used to characterize the interaction between vaginal epithelium, *G. leopoldii* strain (for the strain delineation see Vaneechoutte et al., 2019), and VLY (Garcia et al., 2019).

Cytolysins from *Gardnerella* spp. and *L. iners* share features that attribute them to the CDC family, and it is believed that these toxins have a similar role as their family members during infection *in vivo*. Studies on the functions of VLY and INY in bacterial pathogenesis are still in their infancy. This section focuses on studies that demonstrate the potential role of VLY and INY in the adaptation and survival of bacteria and the progression of vaginal anaerobic dysbiosis.

Ratner et al. reviewed the role of CDCs and other PFTs in bacterial infectious diseases (Los et al., 2013), and identified two main effects of toxins during *in vivo* infection. (1) The primary effect is induced barrier dysfunction of epithelial and endothelial layers via direct and indirect effects caused by cell attack and host immune response at the site of infection, respectively. Among CDCs, these effects are best-known for PLY and SLO (Meehl and Caparon, 2004; Goldmann et al., 2009; Hupp et al., 2012; Wippel et al., 2013). (2) The second effect was disruption of the host immune response by invading host factors, cytotoxicity toward immune cells, or intracellular survival. Impairment of immune defense was studied for LLO, PLY, PFO, and SLO (Marriott et al., 2008; Domon et al., 2016; Osborne and Brumell, 2017; Bhattacharjee and Keyel, 2018). The identified toxin functions involved pathological conditions associated with a bacterial infection.

The study of Los et al. (2013) could not determine whether commensal bacteria or bacteria under non-pathogenic conditions expressed CDCs *in vivo*. Although *L. iners* is not generally considered as a common commensal (Vaneechoutte, 2017b), it is still unclear whether INY is secreted in the healthy vagina (Macklaim et al., 2013) where *L. iners* is abundant. It is hypothesized that INY activity is utilized by *L. iners* to obtain necessary growth nutrients, outcompete other bacteria, and survive under various environmental conditions (France et al., 2016; Petrova et al., 2017; Vaneechoutte, 2017b).

VLY production has been detected in BV-positive women and those with healthy vaginal microbiota (Nowak et al., 2018). The median VLY concentration (1 ng/mL) in vaginal samples with predominant *L. crispatus* is approximately three-fold lower than that in *Lactobacillus*-deficient samples. Healthy microbiota characterized by low pH express minimal VLY activity *in vitro* (Rampersaud et al., 2011). VLY concentrations approximately six-fold higher than those detected *in vivo* caused membrane blebbing but not cell lysis in epithelial cell monolayers (Randis et al., 2013). The formation of membrane blebs induced by PFT-mediated membrane injury is a mechanism of cell protection that promotes the survival of affected cells (Babiychuk et al., 2010; Brito et al., 2019).

Garcia et al. (2019) found that VLY was not essential for intensive growth of the *G. leopoldii* strain AMD on the apical side of a three-dimensional vaginal epithelium model, as VLY does not permeabilize the apical side of vaginal tissue. LaRocca et al. (2014) reported that the hCD59-dependent cytolysins VLY and ILY induced programmed necrosis in mature human erythrocytes. Although erythrocytes are not the primary targets, they may come in contact with cytolysins during menses (Santiago Lopes dos Santos et al., 2012; Schwebke et al., 2014b) and in bacteremia (McCool and DeDonato, 2012; Tankovic et al., 2017). Bacterial growth *in vitro* (including *Gardnerella* spp.) was substantially enhanced by programmed necrosis of erythrocytes. Release of the cytosolic content of erythrocytes may provide required nutrients that lead to a burst in bacterial growth (LaRocca et al., 2014). During menses, the concentrations of *Gardnerella* spp. and *L. iners* increased in the majority of women with healthy vaginal microbiota (Santiago Lopes dos Santos et al., 2012). Menses is the most disturbing factor affecting the stability of vaginal microbial communities (Santiago Lopes dos Santos et al., 2011); therefore, cytolysin-mediated release of nutrients may allow bacteria to overcome this disturbance.

A case report described a *Gardnerella* spp. infection complicated with bacteremia and a toxic-type encephalopathy in a young woman (Tankovic et al., 2017). *G. swidsinskii* strain GV37 isolated from blood cultures produced elevated amounts of VLY *in vitro* compared to that of control strains. The authors hypothesized that high concentrations of VLY *in vivo* can break the blood-brain barrier and affect brain cells.

Treatment of cervical cells with cell-free supernatants of *L. iners* and *G. vaginalis* significantly increased permeability of the cervical epithelial barrier and induced the resulting immune response (Anton et al., 2018). VLY and INY are candidates among the secreted factors that may have induced these effects. However, the authors did not analyze the presence of these toxins in the culture supernatants. The use of *Gardnerella* spp. strains lacking the *vly* gene isolated by our group might help to clarify the effect of cytolysin on cell permeability (Janulaitiene et al., 2018).

The activity of PFTs is related to the host response that defends against bacterial infections (Huffman et al., 2004; Aguilar et al., 2009). Sublytic doses of PFTs result in sublethal numbers of pores, which are perceived by epithelial cells via osmotic stress-induced activation of p38 MAPK signaling (Ratner et al., 2006). The p38 MAPK activation is a conserved response to the disruption of cell membrane integrity caused by many PFTs (Los et al., 2013). Activation of p38 MAPK induces the expression of proinflammatory cytokines that modulate the immune response *in vivo*. Thus, sublytic doses of toxins due to low bacterial density during early infection may function as a signal to epithelial cells to initiate protective immune responses (Ratner et al., 2006). Sublytic doses of INY and VLY induced p38 MAPK activation through phosphorylation in epithelial cells (Gelber et al., 2008; Rampersaud et al., 2011). The mRNA for IL-8 was upregulated after treatment of HeLa cells with VLY (Gelber et al., 2008). Garcia et al. (2019) found that IL-1β was the only cytokine induced by VLY at the basolateral side of vaginal epithelium. Specific IgA antibodies against VLY were detected in 60% of women diagnosed as BV-positive (Cauci et al., 1996); this correlated with IL-8 levels and leukocyte counts in BV-positive and BV-negative women (Cauci et al., 2002).

There have been contradictory reports on cytokine profiles in cervicovaginal lavages of BV-positive women (Mitchell and Marrazzo, 2014). Some studies showed that BV-positive lavages did not have elevated levels of IL-6 and IL-8 and increased neutrophil counts (Cauci, 2004; Donders, 2007; Nowak et al., 2018). By contrast, BV-positive lavages had higher levels of IL-6, IL-8, other proinflammatory cytokines, and chemokines (Hedges et al., 2006; Mitchell and Marrazzo, 2014). The absence of leukocytes might be due to a lack of IL-8 induction in most women with BV, indicating that the host genotype conditions the immune response (Cauci et al., 2002; Mitchell and Marrazzo, 2014).

Several studies have tried to elucidate cytokine induction by individual bacterial species using monolayered or polarized human vaginal epithelial cells that were co-cultured with bacteria or exposed to cell-free supernatants. *L. iners* did not induce an increased production of IL-8 and IL-6 in vaginal and cervical cells (Doerflinger et al., 2014; Anton et al., 2018). However, elevated secretion of these cytokines was detected after exposure of ectocervical cells to *G. vaginalis* cell-free supernatants (Anton et al., 2018). Other studies confirmed that BV-associated bacteria induce cytokine upregulation and lead to the host response, whereas vaginal *Lactobacillus* spp. do not (Mitchell and Marrazzo, 2014). Secreted bacterial factors that modulate the immune response have not been completely determined; however, cytolysin remains as the prime suspect. The absence of inflammation in the lower genital tract is a typical clinical symptom of BV (Donders, 2007). However, BV is linked with clinical conditions that are characterized by inflammation in the upper genital tract, such as pelvic inflammatory disease and cervicitis (Mitchell and Marrazzo, 2014). Non-infectious, inflammation-related preterm delivery is associated with vaginal microbial communities that contain abundant *Gardnerella* spp. (DiGiulio et al., 2015).

## Factors Affecting Cytolysin Expression

The expression of cytolysin genes is affected by environmental changes in oxygen concentration, the impact of other bacteria, biofilm, and planktonic growth phenotypes. Biofilm is a favorable mode of *Gardnerella* spp. growth that confers resistance to environmental factors and contributes to survival (Patterson et al., 2007; Verstraelen and Swidsinski, 2019). The oxygen gradient within a biofilm regulates the distribution and survival of bacterial species (Monds and Toole, 2009; Castro et al., 2019). *Gardnerella* spp. colonize the vagina of many women without inducing signs of BV (Ma et al., 2012; Schwebke et al., 2014a), suggesting that *Gardnerella* spp. may adopt the planktonic style of growth by modulating the gene expression profile (Swidsinski et al., 2014; Castro et al., 2019). Certain *Gardnerella* species or genomic subgroups may have a reduced capacity to adhere to epithelial cells and form a biofilm (Castro et al., 2015; Janulaitiene et al., 2018), probably due to differences in proteins that contribute to and participate in these activities (Harwich et al., 2010; Marín et al., 2018).

Comparative transcriptomic analysis of the *G. leopoldii* AMD strain and several other *Gardnerella* spp. strains demonstrated that the transcription level of the *vly* gene was significantly lower in biofilms than in planktonic cells (Castro et al., 2017). As expected, the transcription of genes responsible for biofilm cell metabolic activity in biofilm cells also was reduced. The authors suggested that VLY was likely not required for the persistence of mono-species biofilm, which represents a long-lasting mode of vaginal colonization. The interactions between *Gardnerella* spp. and other BV-associated bacteria were analyzed using dual-species biofilm assemblies (Castro et al., 2019). Expression of the *vly* gene was significantly upregulated in biofilms when *Gardnerella* was associated with *Enterococcus faecalis* or *Actinomyces neuii*. By contrast, *vly* expression was only slightly upregulated when *Gardnerella* was associated with the BV-related bacteria *Prevotella bivia*. *Atopobium vaginae*, which has most frequently been detected within the *Gardnerella* biofilm matrix, does not affect the *vly* transcription level compared to the mono-species biofilm composed solely of *Gardnerella*. *Mobiluncus mulieris* also did not affect the *vly* transcription level, whereas *Brevibacterium ravenspurgense* repressed *vly* expression. Castro et al. (2019) reported that urogenital bacterial species that were seldom detected in BV had a higher effect on the expression of genes related to virulence than commonly BV-associated *A. vaginae* and *M. mulieris*. One strain of *Gardnerella* spp. isolated from BV-positive women was used in a dual-species biofilm approach. This does not exclude that other species or genomic variants of *Gardnerella* may experience diverse effects. In summary, the *vly* transcript level is lower in *Gardnerella* spp. biofilm than in the planktonic mode of growth. However, certain bacterial species found in the unique dual-species biofilm morphotypes affect *Gardnerella* spp. cells and increase *vly* expression (Castro et al., 2019). The authors proposed that vaginal epithelial cell desquamation characteristic of BV was connected with the increased transcription level of *vly* and VLY-mediated cytotoxicity. By contrast*, L. crispatus*, which is distinctive to a healthy vagina, reduced the cytotoxicity of several *Gardnerella* spp. isolates toward HeLa vaginal epithelial cells by repressing *vly* expression (Castro et al., 2018).

Application of the meta-transcriptomic approach revealed that INY was upregulated six-fold and VLY was upregulated 256-fold in BV compared to a healthy state (Macklaim et al., 2013). Mitchell and Marrazzo (2014) reported that it was not clear whether these changes occurred due to strain differences or because the microbial environment reflected the gene expression levels. These studies require further investigation and analysis to detect differences in the expression profiles of cytolysins isolated from a large number of vaginal samples.

## Discussion and Perspectives

CDCs have evolved a sophisticated mechanism to punch a hole in host membranes and invade the cells. Cytolysin-mediated disruption of membrane integrity of the cell barrier leads to various consequences to the host, from the direct cell death to impaired cellular functions. Research challenges still remain, such as determining the structure-function relationships *in vivo*, although many issues regarding the structure of CDCs and their pore-forming activity have been elucidated. Some CDC activities are accomplished by pore-independent pathways. All these activities promote growth and invasion of bacterial pathogens; therefore, CDCs are accepted as important virulence factors contributing to the pathology of infectious diseases. Bacterial species that are not regarded as traditional human pathogens also produce cytolysins, including *Gardnerella* spp. and *L. iners*. Studies to elucidate the role of *Gardnerella* as a pathogen have been performed for more than 60 years. In the recent past, this taxon was underrated, but has been recently revived by the definition of vaginal *Gardnerella* polymicrobial biofilm as a characteristic of BV, and identifying species and genomic groups with potentially diverse roles in health and disease. Future work will continue to delineate the importance of *L. iners*.

This review provides an overview of structural and activity characteristics of the cytolysins VLY and INY (Table 1). The environmental pH, accessibility of membrane cholesterol, and cytolysin concentration may affect the activity and function of these CDCs on eukaryotic cells. However, many issues remained unsolved. The membrane composition that is more prone to cytolysin binding remains to be determined, which may condition the specificity to the target during infection and in a commensal state. The impact of hCD59 on some characteristics of VLY activity needs to be fully elucidated because the majority of studies on synthetic membranes did not include this receptor. Knowledge of the genetic regulation of *vly* and *iny* expression is lacking. It is unclear whether non-hCD59-responsive INY is sensitive to other binding targets besides cholesterol. *Gardnerella* spp. and *L. iners* cytolysins are considered as virulence factors by analogy with other CDCs, although their particular roles *in vivo* are unknown. This report also provides insights into the predicted and hypothetical functions of VLY and INY *in vivo* (Table 1). Prospectively, the effects of cytolysins *in vitro* should be studied by the inclusion of different *Gardnerella* species and *L. iners* strains, and strains lacking *vly* and *iny* genes. Models that more closely resemble *in vivo* conditions are needed to unravel the relevance of cytolysins to the pathogenesis of BV. The polymicrobial nature of BV is technically challenging for studying the effects of cytolysins, as it is an experimentally evident influence of neighboring vaginal bacteria on the virulence genes. It is crucial to study the role of cytolysins in the context of host microbiota, its fluctuations, and disturbance that condition the virulence potential of bacterial species.

**Table 1:** Summary of vaginolysin and inerolysin characteristics.

**Characteristic**	**Vaginolysin**	**Inerolysin**
CDC-producing bacteria	*Gardnerella* species	*Lactobacillus iners*
Presence of the CDC coding gene	Majority of isolates	Prevalence among isolates is not well known
Mode of action	Form β-barrel pores in the membrane	
Structural features	Display a four-domain structural organization; possess the key sequence motifs characteristic of CDCs	
Receptor	Human CD59 or membrane cholesterol	Membrane cholesterol
Pore-forming mechanism	Bind to the cholesterol-rich membranes, oligomerize into large oligomeric complexes (rings and arciform structures), undergo conformational changes leading to membrane perforation and pore formation	
Membrane cholesterol	Indispensable for the pore-forming activity	
Membrane cholesterol concentration required for CDC activity	>40 mol%	>20 mol%
pH activity range	5.0–7.5	4.5–6.0
Effect (1): lytic CDC concentrations	Direct cell damage: formation of water-filled pores in the target cellular cholesterol-containing membranes	
Effect (2): sublytic CDC concentrations	Non-lytic pores modulate signaling cascades: induce p38 MAPK activation and cytokine expression	
Effect (3): independent of pore formation	Not known	Not known
Pore size on synthetic cholesterol-rich membranes	Dominate large pores (ring structures)	Dominate smaller-size pores (aciform structures)
Presence in healthy vagina	Yes	Not known
Role in healthy vagina	VLY is not active	Not known
Presence in BV	Yes	Yes
Plausible role in BV	VLY-mediated cytotoxicity results in vaginal epithelial cell desquamation	INY-mediated disruption of the host cell integrity to obtain nutrients necessary for growth, outcompete other bacteria, and survive in the vaginal environment

## Author Contributions

The author confirms being the sole contributor of this work and has approved it for publication.

### Conflict of Interest

The author declares that the research was conducted in the absence of any commercial or financial relationships that could be construed as a potential conflict of interest.
